# Illuminating outcomes: Diode laser management of an uncommon leukoplakia of tongue - A case report

**DOI:** 10.6026/973206300211490

**Published:** 2025-06-30

**Authors:** Ramnath Elangovan, Keerthika Varadhan, Kowsalya Nallathambi, Dinesh C. Maganti, Danilo Milanes Zambrano, Anitha Maganti

**Affiliations:** 1 Department of Periodontics and Community Dentistry, School of Dentistry, University of Rwanda, Kigali, Rwanda, East Africa; 2 Department of Periodontics, Adhiparasakthi Dental College and Hospital, Melmaruvathur, Tamilnadu, India; 3 Department of Paediatric Dentistry and Orthodontics, School of Dentistry, University of Rwanda, Kigali, Rwanda, East Africa; 4 Department of Stomatology, Hospital La Croix du Sud, Kigali, Rwanda, Kigali, Rwanda, East Africa

**Keywords:** Leukoplakia, diode laser, potential malignant disorders, laser ablation, low level laser therapy

## Abstract

Oral leukoplakia exists as a potentially cancerous tissue condition which frequently develops because of tobacco habit and various associated risks. The treatment approach with diode laser on leukoplakia was completed in a 61-year-old male who had a lateral tongue abnormality. A 940 nm diode laser applied in gated pulse mode performed the lesion removal which created minimal intraoperative bleeding and fast postoperative recovery with no sign of disease recurrence. The examination of tissue under a microscope showed hyperplastic stratified squamous epithelium together with dysplasia. Diode laser provides a powerful therapeutic option with minimal invasiveness for treating oral leukoplakia when used on medically vulnerable patients.

## Background:

Oral leukoplakia represents one of the cancer-containing conditions which develop in oral mucosal tissue. The condition exists as "a set of solid oral white tissue marks which cannot match any different diagnostic identification." Oral leukoplakia appears as white oral patches known as white plaques throughout the oral cavity due to smoking habit. Any form of tobacco usage activity from cigars to cigarettes and beedis and pipes functions as a risk factor. A variety of factors such as alcohol use, chronic irritation, oral galvanism generated by restorations, bacterial infections, sexually transmitted diseases particularly syphilis, deficient micro-nutrients, viral infections, hormone imbalances and ultraviolet exposure function as synergistic risk elements. Oral leukoplakia presents multiple idiopathic roots that stem from complex causative factors [1]. Tobacco products used either as smoke or non-tobacco acknowledge as the most widespread risk factor [2]. Also, the consumption of areca (betel) nut preparations, as well as snuff and other types of smokeless tobacco, poses a considerable risk in many parts of the world (particularly in South and Southeast Asia) [3].

Research has established a connection between chronic candidiasis forming leukoplakia mainly through the development of nonhomogeneous leukoplakia [4]. The high nitrosation capability of specific candidal cells may lead to the synthesis of endogenous nitrosamines [5]. In different cultures people retain tobacco smoke from burning devices inside their mouths producing multiple oral mucosal injuries including leukoplakia. Among societies where tobacco exists in different forms the presence of such leukoplakia leads to a 19-fold increase in cancer development risk [6].

## Case report:

A male patient aged 61 visited the Department of Periodontics due to a 3-month presence of whitish growth on his tongue. He describes the presence of itching sensations on the affected area. The patient faced hypertension and diabetics within his medical background along with having regular medication for 12 years. Oral dental work that included scaling happened more than three years ago while smoking ended five years ago. The extraoral evaluation resulted in no notable findings. No swollen lymph nodes could be felt in the regional areas. The intraoral appearance exhibits a smooth white growth with a 2 x 11 cm size and flat-wrinkled surface. On palpation it is non-tender, scrapable and smooth in consistency. Local anaesthesia received application before the physician conducted an excisional biopsy. The dental practitioner performed the excisional procedure on the growth using diode laser equipment at Gated pulse mode (Indilase 980nm, 1w). The surgical assistant employed the tissue pliers for handling the growth. A pathologist sent the removed tissue to histological testing after placing it in 10% formalin solution. The patient experienced peace throughout the procedure while no bleeding occurred and the patient required no sutures. Doctors decided not to administer any antibiotics following the procedures. Healthcare providers gave instructions to patients about taking analgesics when needed. The patient returned one week later, and healing presented as exceptional.

## Steps:

[1] The Lesion is marked with hematoxylin pencil including a small amount of normal tissue.

[2] Under adequate Local anesthesia [1:80,000} a local infiltration given in the dorsal surface of the tongue.

[3] Excision of the lesion done using 940 nm Diode laser with the power setting of 1W in Gated Pulse mode and sent for biopsy.

[4] Patient was recalled after one and four weeks for assessment of healing.

[5] Three months post-operative image showed 100 percent of surgical site is healed.

## Histopathology:

H & E-stained section under 40xmagnification reveals fibrocellular connective tissue area with an overlying hyperplastic stratified squamous epithelium with acanthoesis, thick layer of orthokeratin and prominent granulosis. The connective tissue is interspersed with capillaries of varying sizes with extravasated RBC'S and mild inflammatory cell infiltration seen adjacent the epithelium. Deeper connective tissue shows cross sections & longitudinal sections of muscles and adipose tissue.

## Discussion:

Based on the direct link between clinical observations and microscopic assessment the professional concluded the patient suffered from Leukoplakia. Oral leukoplakia functions as a disease that has transformation potential into malignancy. The occurrence of malignant transformation in the Indian population reaches 3% as reported in studies [7]. Evidence shows that Leukoplakia presents higher potential for malignant development with studies demonstrating that the rate of malignant change exists between 0.13% and 36.4% [8]. Lateral tongue lesions found in older males from India suppose the highest potential for cancer development according to Vander Waal and Arduino [7, 8]. Tobacco usage together and alcohol intake with betel tobacco consumption and also genetic defects serve as potential origins for the development of OLK [9-10]. The current surgical experience along with postoperative recovery required minimal pain from this patient. The healing process of the wound showed satisfactory results because LASER treatment was applied. The areas around the dentogingival junction along with the nonkeratinized lateral border of the tongue experience fast cell turn-over because these areas easily develop microbial biofilms and face damage from mechanics as well as surface tobacco usage that advance cancerous transformations [11]. Our clinical observations demonstrate that OED patients develop malignant transformations affecting 22% of cases over 5 years of follow-up. A patient who does not smoke and has a tumor located on the lateral side of the tongue with heterogeneous appearance faces a minimum 40% possibility of malignant progression in five years. Nonsmokers suffered from malignant tumor development in rates 7.1 times higher than heavy tobacco users [12]. The first main category shows congenital non-scrapable white oral lesions which appear early in life while displaying a family history of such involvement. A diagnosis of Leukoedema should occur after noting the stretching effect dissolves the white patch in patients who both smoke and exhibit lesions on their buccal mucosa. Alpha arthroplasty represents the second significant type of oral white lesions because they can be easily brushed off from the surface. The group's structural lesions include mucosal burns as well as morsicatio and pseudomembrane of ulcers which doctors can detect from both clinical exams and patient history collections. Pseudomembranous candidiasis occurs frequently among newborns without causing serious concerns but becomes a concern for adults and elderly people due to systemic or local predisposing elements including severe illnesses or oral microbiota changes. White keratotic lesions with particular patterns constitute the third principal oral white lesion category but cannot be easily distinguished from other conditions by clinical or microscopic findings. The group consists of oral lichen planus together with lichenoid contact reaction and drug-induced lichenoid reaction as well as graft against host disease [13]. This treatment illustrates why less invasive surgical practices present significant advantages to patients who have pre-exiting health conditions and are elderly. Laser surgery provides three primary advantages which comprise reduced operating bleeding and minimal postoperative symptoms in addition to accelerated recovery time [14]. The patient received better healing outcomes from treatment with low-intensity lasers through photo-biomodulation because these lasers activate cell processes to improve tissue restoration. Clinical data from the follow-up period demonstrates that the selected treatment approach was successful because the patient experienced no recurrence during six months [15]. Laser offers many advantages over other conventional methods including lesser postoperative pain and swelling, bloodless dry operating field and increased patient comfort and requires minimal local anaesthesia [16].

## Conclusion:

Initiating early diagnosis of premalignant lesions requires the immediate involvement of doctors specializing in oral medicine together with dentists thereby beginning the treatment immediately to minimize the pathological changes even when symptoms are absent because it results in reduced cancer cell transformation possibilities after primary etiology removal. This case report underscores the efficacy of early diagnosis and diode laser intervention in managing leukoplakia, highlighting its role in minimizing recurrence and enhancing patient outcomes.
